# Investigating the field-dependence of the Davis model: Calibrated fMRI at 1.5, 3 and 7 T

**DOI:** 10.1016/j.neuroimage.2015.02.068

**Published:** 2015-05-15

**Authors:** Hannah V. Hare, Nicholas P. Blockley, Alexander G. Gardener, Stuart Clare, Daniel P. Bulte

**Affiliations:** FMRIB Centre, Nuffield Department of Clinical Neurosciences, University of Oxford, Oxford, UK

**Keywords:** Calibration, BOLD, Hypercapnia, MRI, Field strength

## Abstract

Gas calibrated fMRI in its most common form uses hypercapnia in conjunction with the Davis model to quantify relative changes in the cerebral rate of oxygen consumption (CMRO_2_) in response to a functional stimulus. It is most commonly carried out at 3 T but, as 7 T research scanners are becoming more widespread and the majority of clinical scanners are still 1.5 T systems, it is important to investigate whether the model used remains accurate across this range of field strengths. Ten subjects were scanned at 1.5, 3 and 7 T whilst performing a bilateral finger-tapping task as part of a calibrated fMRI protocol, and the results were compared to a detailed signal model. Simulations predicted an increase in value and variation in the calibration parameter M with field strength. Two methods of defining experimental regions of interest (ROIs) were investigated, based on (a) BOLD signal and (b) BOLD responses within grey matter only. M values from the latter ROI were in closer agreement with theoretical predictions; however, reassuringly, ROI choice had less impact on CMRO_2_ than on M estimates. Relative changes in CMRO_2_ during motor tasks at 3 and 7 T were in good agreement but were over-estimated at 1.5 T as a result of the lower signal to noise ratio. This result is encouraging for future studies at 7 T, but also highlights the impact of imaging and analysis choices (such as ASL sequence and ROI definition) on the calibration parameter M and on the calculation of CMRO_2_.

## Highlights

• Calibrated BOLD was performed at three common magnetic field strengths.
• Simulations predicted increased value and variation in M with field strength.
• Relative oxygen metabolism was consistent between 3 and 7 T but larger at 1.5 T.
• The disagreement at 1.5 T is likely due to the lower SNR of ASL acquisitions.
• Two ROI methods were compared, which gave similar rCMRO2 but different M results.

## Introduction

Gas calibrated functional magnetic resonance imaging (fMRI) has emerged as a promising tool to non-invasively measure stimulus evoked changes in the cerebral metabolic rate of oxygen consumption (CMRO_2_) (Davis et al., 1998; Hoge, 2012). Not only is this more directly physiologically relevant than measuring only the blood oxygen level-dependent (BOLD) signal, but it has also been shown to be more consistent between subjects and between scanning sessions (Krieger et al., 2014).

Calibrated fMRI is most commonly performed at 3 T, but with the emergence of 7 T research systems there is interest in translating the method to higher field strengths. It is clear that as a physiological parameter CMRO_2_ should not be affected by the field strength at which it is measured. However, calibrated fMRI relies on a simple model of the BOLD signal known as the Davis model (Davis et al., 1998) and it is unclear how translation to different field strengths affects its accuracy. Due to the continuing clinical dominance of 1.5 T scanners, we were also interested in revisiting the use of this lower field strength, enabling a three-way comparison to be performed. Simulations of the calibrated fMRI experiment suggest that the method is feasible at these field strengths, so long as the Davis model parameters are adjusted to reflect the altered BOLD sensitivity (Blockley et al., 2013; Griffeth et al., 2013). It has also been predicted that systematic error may be increased at 7 T compared with lower field strengths (Griffeth et al., 2013). Experimental confirmation of these findings has so far not been performed.

The aim of this study was to carry out a direct comparison of the hypercapnia calibrated fMRI method at 1.5, 3 and 7 T, in order to test the level of agreement in the measured estimates of relative CMRO_2_ across field strengths. Detailed simulations of the BOLD signal were performed to predict general trends in the calibration parameter M across field strengths, and to provide a basis for interpreting the experimental results. Implicitly this study is also an investigation into the robustness of the Davis model itself, which was originally developed for use at 1.5 T, and how well it translates across field strengths.

## Theory

The Davis model (Eq. (1)) describes the extravascular BOLD signal as a function of changes in cerebral blood flow (CBF) and CMRO_2_ (Davis et al., 1998; Hoge et al., 1999). Subscript 0 variables represent baseline values. Changes in CBF are related to changes in cerebral blood volume (CBV) via the Grubb exponent α (Grubb et al., 1974). This physiological interpretation of *α* was retained and was therefore set independent of field strength as 0.2 (Chen and Pike, 2009, 2010). The BOLD signal is related to underlying changes in susceptibility via the exponent *β*, which varies with vessel size and also with field strength.

(1) $$\frac{\mathrm{\Delta BOLD}}{{\mathrm{BOLD}}_{0}}=M\left\{1-{\left(\frac{{\mathrm{CMRO}}_{2}}{{\mathrm{CMRO}}_{2}|{}_{0}}\right)}^{\beta }{\left(\frac{CBF}{{CBF}_{0}}\right)}^{\alpha -\beta }\right\}$$

In the Davis model the calibration parameter M is defined as TE × A × CBV_0_ × [dHb]_*v*0_^*β*^, where TE is the BOLD-weighted echo time, A is a proportionality constant which depends on field strength and tissue properties, CBV_0_ is the baseline CBV and [dHb]_*v*0_ is the baseline venous deoxyhemoglobin concentration of the blood (Hoge et al., 1999). Therefore, the calibration parameter is expected to vary with inter-subject or inter-session variations in physiology and tissue type. Hence it is necessary to calculate M during each scan session. This is most commonly achieved by applying an isometabolic stimulus, such as mild hypercapnia, during which CMRO_2_ is assumed to remain constant (Hoge, 2012). Eq. (1) can then be rearranged to estimate M given measurements of the changes in CBF and BOLD signal acquired during the hypercapnic challenge.

The definition of M also describes a dependence on the BOLD weighted TE. Since many different values of TE have been used throughout the literature it is often instructive to be able to scale the calibration parameter to the optimal BOLD echo time for each field strength, which in this study were considered to be 50/35/25 ms at 1.5/3/7 T, respectively (van der Zwaag et al., 2009). To do this we assume that A has no dependence on TE, and multiply the experimental M by the ratio (optimal TE/actual TE).

## Methods

### BOLD signal simulations

A detailed model of the BOLD signal (Griffeth and Buxton, 2011) was used to simulate the hypercapnia calibration experiment, with the aim of predicting general trends in M across field strengths and to aid in interpreting experimental results. The original detailed signal model was designed to simulate the BOLD signal at 3 T. In order to extend the model to 1.5 and 7 T a few modifications were made, which are detailed below. In brief, the basic model consists of a volume-weighted sum of arterial (*a*), capillary (*c*) and venous (*v*) intravascular compartments and a single extravascular (*e*) compartment.

(2) $$S=\left(1-V_{a}-V_{c}-V_{v}\right)S_{e}+V_{a}S_{a}+V_{c}S_{c}+V_{v}S_{v}$$

The signal contributions were summed according to Eq. (2) under baseline and hypercapnic conditions and combined to simulate the BOLD signal change (see appendix of Griffeth and Buxton (2011)). Extravascular signals (*S_e_*) were modelled using the results of numerical simulations for two vessel scales to reflect the different signal characteristics of capillaries (*β* = 2) compared with arteries and veins (*β* = 1) (Ogawa et al., 1993). These signals were added to the baseline *R*_2_^⁎^ of the extravascular tissue space (*R*_2E_*(0)). Intravascular signal contributions (*S_a_*, *S_c_*, *S_v_*) have been described by empirical measurements of the blood transverse relaxation rate *R*_2_^⁎^ as a function of oxygenation and haematocrit (Zhao et al., 2007). Models of both extravascular and intravascular signal enabled the effects of blood oxygenation and haematocrit to be simulated. Blood was distributed to each of the compartments as a fraction (Ω) of the total CBV (*V_T_*), e.g. *V_a_* = Ω*_a_ V_T_*. Relative volume fractions were set as Ω*_a_* = 0.2, Ω*_c_* = 0.4, and Ω*_v_* = 0.4. However, the model described thus far is only applicable at 3 T.

The following modifications were made to enable simulations to be performed at all three field strengths used in this study. The existing empirical measurements of blood *R*_2_^⁎^ were replaced by the results of a multi-field relaxometry experiment (Blockley et al., 2008). Due to the limited 7 T relaxometry literature it was only possible to generalise *R*_2_* for oxygenation and not haematocrit. The quadratic relationship of *R*_2_^⁎^ on oxygenation was retained, i.e. *R*_2_^⁎^ = *C*_1_ + *C*_2_ × (1 − *Y*)^2^, where *C*_1_ and *C*_2_ are field strength specific constants and *Y* is the fractional blood oxygenation. Therefore, unlike the previous model where *R*_2_* was modelled as a function of oxygenation and haematocrit, the intravascular signal dependence on oxygenation in the present study was described for a fixed haematocrit (Hct = 0.44) at all field strengths. Whilst this limits the ability of the model to test the effect of inter-subject physiological variability, it enables general trends in M to be investigated across a range of field strengths. Baseline extravascular tissue *R*_2_^⁎^ values (*R*_2E_*(0)) were assigned field-specific values based on relaxometric measurements made at each field strength (van der Zwaag et al., 2009). BOLD echo times were set to match the experimental acquisition. These parameters are summarised in Table 1.

Simulations were initially performed for the baseline physiological state of a hypothetical average healthy individual. Haematocrit was set to be 0.44 (McPhee and Hammer, 2009), oxygen extraction fraction (OEF) was set at 0.4 (Hatazawa et al., 1995) and total CBV for grey matter was set as 0.05 (Perlmutter et al., 1987). Hypercapnia calibration was simulated as a 30% increase in CBF with no change in CMRO_2_. The BOLD signal response to this challenge was simulated using the detailed BOLD signal model. The BOLD and CBF changes were then combined with Eq. (1) to calculate M at each field strength using an *α* of 0.2 (Chen and Pike, 2009, 2010) and *β* values of 1.5/1.3/1.0 at 1.5/3/7 T respectively, consistent with the literature (Bulte et al., 2009; Davis et al., 1998; Driver et al., 2012). Estimates of M are dependent on the baseline physiological state of the individual and it is therefore expected to vary across the population. To test how this variability is affected by different field strengths, M was estimated for BOLD signals generated with several values for baseline CBV and OEF. Perlmutter et al. (1987) recorded a mean CBV of 0.05 ± 0.01. Extending the range to 2 standard deviations from the mean, limits of 0.03 and 0.07 were also simulated. Similarly, resting OEF values of 0.3, 0.4 and 0.5 were investigated (Hatazawa et al., 1995) whilst keeping CBV constant at 0.05. Simulated M values were linearly scaled to the optimal TE values for each field strength for comparison purposes.

### MRI parameters

Subjects were scanned on 1.5 T Avanto, 3 T Verio and 7 T systems (Siemens Healthcare, Erlangen, Germany) with 12-channel (1.5 T) and 32-channel (3 and 7 T) head coils. Scans were carried out on separate days to minimize the effects of fatigue and habituation. Because specific absorption rate (SAR) was anticipated to be a limiting factor on sequence design at 7 T, a pulsed (rather than pseudo-continuous) ASL sequence was implemented to measure CBF (Alsop et al., 2015). Flow-sensitive alternating inversion recovery (FAIR) (Kim, 1995) was chosen to minimize the effects of B_1_ inhomogeneity (Gardener et al., 2009), and the QUIPSS II scheme (Wong et al., 1998) was used to improve quantification of perfusion.

A single echo at 17 ms provided sufficient signal to noise ratio (SNR) for both CBF and BOLD analysis at 3 and 7 T; a dual echo version of the same sequence was implemented at 1.5 T with echoes at 17 and 50 ms to ensure sufficient BOLD contrast. All other imaging parameters were kept constant across scanners. Six slices were acquired (limited by SAR at 7 T) with an echo-planar imaging (EPI) readout and were placed axially to cover the motor cortex. For consistency across scanners, no acceleration methods (such as parallel imaging or partial Fourier) were used. Bandwidth was set to 3004 Hz/Px, inversion times were TI_1_ = 700 and TI_2_ = 1800 ms, and repetition time was 3 s. Note that in pulsed ASL the bolus duration is fixed by TI_1_ and the effective post-labelling delay (PLD) is given by TI_2_–TI_1_ (Alsop et al., 2015), so 1100 ms in this experiment. It is common to use shorter PLDs in pulsed ASL compared to (pseudo-)continuous implementations to compensate for the reduced SNR inherent in pulsed ASL. Although this may result in incomplete delivery of tagged blood to the imaging slices, it is also more suited to gas-calibrated experiments, where arterial arrival times are shortened during hypercapnia. Voxel size was 4.1 × 4.1 × 5.0 mm^3^ with a 1 mm slice gap in order that SNR of ASL data at 1.5 T did not become prohibitively low. FAIR labelling used a 60 mm selective and a 260 mm non-selective slab at all field strengths.

### Functional and respiratory tasks

**Fig. 1:**
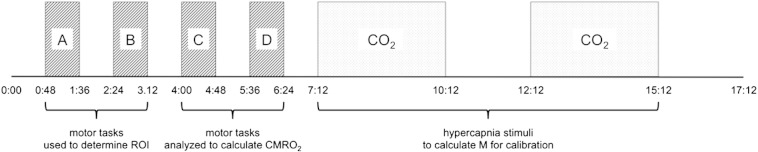
Diagram showing timing of stimuli. Total paradigm length 17 min 12 s.

A bilateral finger tapping motor task was chosen to easily allow a consistent implementation across scanner suites. Subjects were given audio cues over the intercom systems and were instructed to perform 4 blocks of self-paced finger tapping (48 s ON, 48 s OFF). This was followed by 2 blocks of hypercapnia (3 min duration, each followed by 2 min of air), as shown in Fig. 1. Subjects were instructed to perform the motor task at a fast but comfortable rate, and these instructions were repeated prior to each scan session. Performance of the task was monitored from the control room and all subjects were observed to fully cooperate.

Gas delivery and sampling was achieved through a nasal cannula (dual Nare, Flexicare, Mountain Ash, UK) in conjunction with a CO_2_ gas analyzer (CO2 100C Biopac Systems, Goleta, CA, USA). A 10% CO_2_/21% O_2_ gas mixture with balance nitrogen was delivered, which mixed with room air at a ratio of approximately 1:1, resulting in an ~ 5% CO_2_ stimulus. In order to minimize subject awareness of the hypercapnia stimulus, medical air (21% O_2_ with balance nitrogen) was delivered during periods of air breathing, including during the motor tasks.

### Analysis

Data were analyzed using the FMRIB Software Library (FSL) and the fMRI Expert Analysis Tool (FEAT) (Woolrich et al., 2009). Preprocessing consisted of motion correction (Jenkinson et al., 2002), brain extraction (Smith, 2002) and fieldmap correction (Jenkinson et al., 2012). Highpass filters of 10 and 300 s were applied for ASL and BOLD respectively, and no spatial smoothing was applied. The following contrasts were input as part of a general linear model within FEAT: (1) tag-control signal, modelled as a square on/off shape, which describes baseline perfusion; (2) BOLD response to motor tasks A + B, with a haemodynamic response function modelled as a gamma convolution with mean lag 6 s, standard deviation 3 s; (3) BOLD response to motor tasks C + D, as above; (4) BOLD response to hypercapnia, modelled with a gamma convolution with mean lag 42 s, standard deviation 30 s to account for the delay between switching to the CO_2_ mixture and the cerebrovascular response to increased arterial partial pressure of CO_2_ in the brain; (5–7) blood flow responses to motor tasks A + B, C + D and hypercapnia, modelled as interactions between contrasts (1) and the three BOLD responses, respectively. In this context, the term “interaction” refers to the fitting of the product of two existing contrasts, and may be modelled in FEAT simply by checking the relevant tick box. To avoid circular analysis (Kriegeskorte et al., 2009), data from motor tasks A + B were used to create a region of interest (ROI), and only data acquired during motor tasks C + D were analyzed to quantify response to motor stimuli (see Fig. 1). Other combinations of block-wise analysis were investigated (e.g. A + D for ROI definition, B + C for data analysis), and this choice was found to have no substantial impact on the results.

Two methods were used to create motor ROIs. First, a “BOLD only” ROI was defined as the 40% of voxels with the highest uncorrected voxel-wise z-stats in response to motor tasks (A + B only, see Fig. 1). This cutoff was used in place of setting a z-stat threshold directly as it was more consistent between subjects and particularly across field strengths. Similarly, a grey matter (GM) mask was created from the 40% of voxels with the most significant baseline tag-control signal difference. Multiplication of the BOLD ROI and GM mask produced a “BOLD/GM” motor ROI. The creation of “CBF only” and “BOLD/CBF overlap” ROIs was not possible due to the low SNR of ASL data, particularly at 1.5 T.

Further analysis was performed on coefficient of parameter estimate (COPE) outputs of the FEAT analysis in MATLAB (MathWorks, Natick, MA, USA). BOLD signal responses were normalized with respect to the mean signal over the entire time course, and CBF responses were normalized to the baseline perfusion signal, as output by the tag-control COPE. Voxels with a BOLD response to either motor or hypercapnia stimuli < 0 or > 0.10, or a CBF response < 1 (no change) or > 3 (+ 200% relative to baseline), were assumed to be noise or to contain significant fractions of white matter or cerebrospinal fluid, and were excluded from further analysis. Mean values within the remaining voxels for BOLD and CBF responses to motor tasks (C + D) and to hypercapnia were used to calculate M and relative CMRO_2_ on a per subject basis, according to Eq. (1), with the assumption that mild hypercapnia does not alter CMRO_2_. The Grubb exponent *α* was set at 0.2 for all field strengths (Chen and Pike, 2009, 2010), and *β* values of 1.5/1.3/1.0 were used at 1.5/3/7 T respectively (Bulte et al., 2009; Davis et al., 1998; Driver et al., 2012). Finally, M values at 3 and 7 T were scaled to optimal echo times for comparison purposes.

Relative changes in CMRO_2_ induced by the motor tasks were compared across field strengths using the one sample paired t-test, where the null hypothesis that measured CMRO_2_ changes are independent of field strength was rejected if p < 0.05. Bland–Altman diagrams were used to investigate any systematic biases between field strengths. ASL image SNR was calculated from a single resting tag-control subtracted time point, as the mean ASL signal within the BOLD/GM ROI divided by the standard deviation in a non-brain ROI. In order to avoid unfair bias, noisy voxels were *not* excluded from the BOLD/GM ROI for this calculation.

## Results

### BOLD signal simulations

**Fig. 2:**
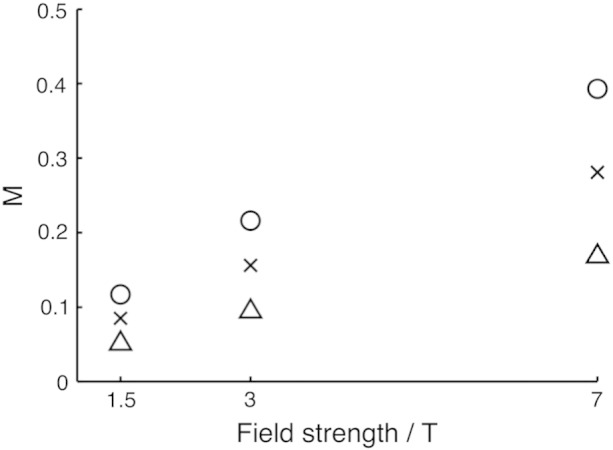
Simulated M values produced using the detailed BOLD signal model. Triangles, crosses and circles represent the predicted M values for individuals with a blood volume fraction of 0.03, 0.05 and 0.07 respectively.

Simulations of hypercapnia calibration using a CBV of 0.05 and the actual experimental TE's used predicted M values of 0.085 at 1.5 T, 0.076 at 3 T and 0.191 at 7 T. When scaled to the optimal TE values, M was predicted to be 0.085 at 1.5 T, 0.156 at 3 T and 0.281 at 7 T. These optimal values are plotted in Fig. 2 as crosses; circles and triangles represent the predicted M values for ± 2 standard deviations for physiological CBV values respectively. The simulations predict that variation in resting CBV leads to a greater range of M values at higher magnetic field strengths. Similar increases in the range of M values with field strength were observed when the range of baseline total OEF was investigated (data not shown).

### Experiment

**Fig. 3:**
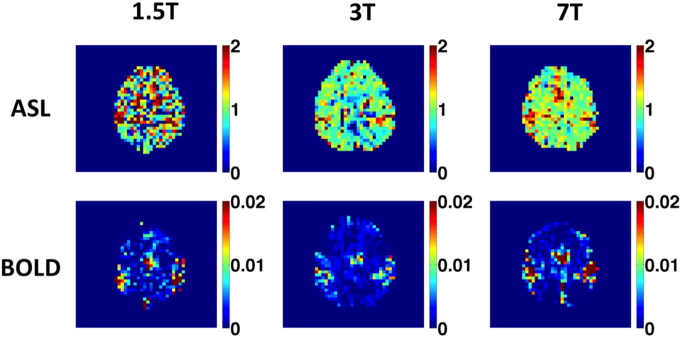
ASL and BOLD responses to motor tasks for one representative subject. ASL units are normalized to baseline, as are BOLD signal increases. Note that no smoothing has been applied.

Ten consenting subjects (3 females, mean age 29 ± 6 years) were successfully scanned on 1.5, 3 and 7 T systems. Examples of fractional BOLD and CBF responses to motor tasks are shown in Fig. 3. Group average responses to hypercapnia and motor tasks are summarised in Table 2. End-tidal CO_2_ levels were monitored throughout the course of the experiments; however, the equipment performed poorly and the resultant traces were deemed unreliable, and thus are not reported here. Although unconfirmed, the authors have no reason to believe that end-tidal CO_2_ modulations were inconsistent between the three scan suites. The BOLD response appears smallest at 3 T because of the shorter than usual echo time (TE = 17 ms), whereas the echo times used at 1.5 and 7 T were close to the field-optimized values. Nevertheless, the BOLD signal at this shorter echo time of 17 ms at 3 T was easily sufficient for this analysis; hence a second echo was not acquired. Changes in CBF are consistent between 3 and 7 T but are substantially higher at 1.5 T for both stimuli and both ROIs.

**Fig. 4:**
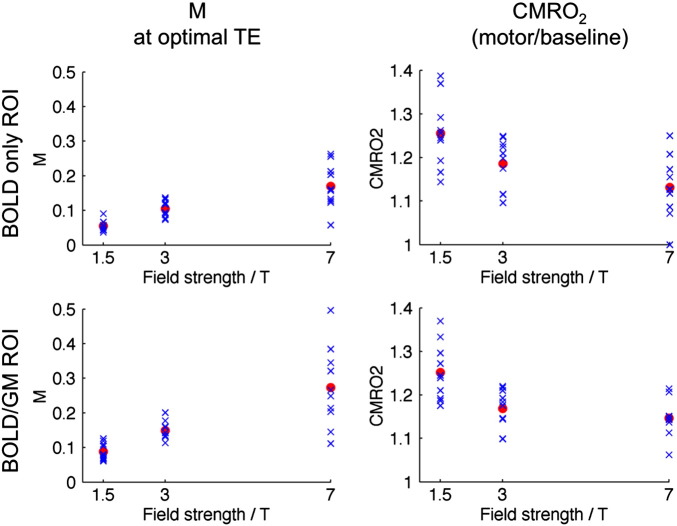
M and relative CMRO_2_ as a function of field strength. Individual subjects are marked by blue crosses, group means by red circles.

Fig. 4 shows how M and relative CMRO_2_ to motor tasks vary with field strength, where each blue cross represents an individual subject, and group means are indicated by red circles. The BOLD/GM ROI produces consistently higher M estimates than the BOLD only ROI. There is a much larger standard deviation in M values at 7 T than at 1.5 or 3 T; however this variability does not propagate to CMRO_2_, where the largest inter-subject standard deviation was always seen at 1.5 T.

One sample paired t-tests were carried out to determine whether relative CMRO_2_ changes were consistent between field strengths. For the BOLD/GM ROI analysis, 1.5 T and 3 T results were shown to be inconsistent (p = 0.022). Similarly, the null hypothesis was rejected when comparing 1.5 T and 7 T (p < 0.001). However, 3 T and 7 T did not yield significantly different results (p = 0.166). When considering relative CMRO_2_ in the BOLD only ROI, only 1.5 T vs. 7 T reached statistical significance (p = 0.001).

**Fig. 5:**
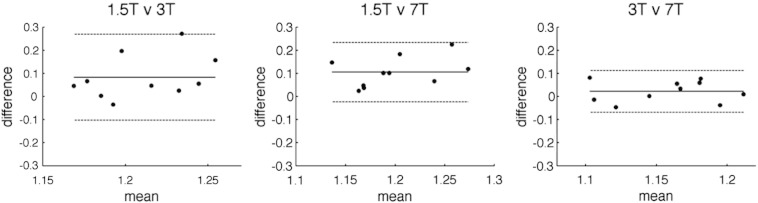
Bland–Altman plots comparing relative CMRO_2_ to motor tasks (BOLD/GM ROI) at different fields. In all diagrams, mean differences are shown as solid lines and 95% confidence intervals as dashed lines.

The Bland–Altman diagrams in Fig. 5 show graphically that the relative CMRO_2_ to motor tasks as estimated by the Davis model at 3 and 7 T are in good agreement. However, they are consistently higher when carried out at 1.5 T, compared with either 3 or 7 T, by an average of ~ 10% (using the BOLD/GM ROI).

**Fig. 6:**
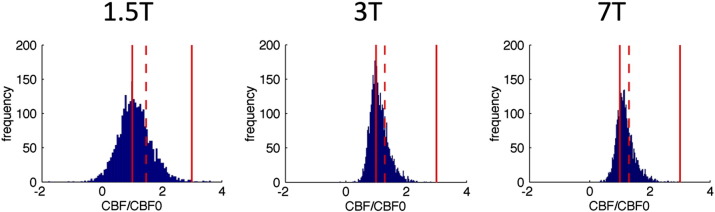
Histograms of voxel-wise blood flow response to motor tasks (C + D only), pooled from all 10 subjects' BOLD/GM ROIs. Solid red lines indicate cutoff conditions beyond which voxels were excluded from further analysis; dashed red lines indicate means of remaining voxels.

CBF responses to motor tasks (Fig. 6) and hypercapnia (data not shown) had a significantly broader distribution at 1.5 T than at 3 or 7 T. After applying voxel exclusion criteria (illustrated by solid red lines in Fig. 6), the mean of the remaining voxels (indicated by the dashed lines) was larger at 1.5 T than at higher fields. For illustrative purposes Fig. 6 includes data from all 10 subjects, but the same trends were seen at the single subject level.

**Fig. 7:**
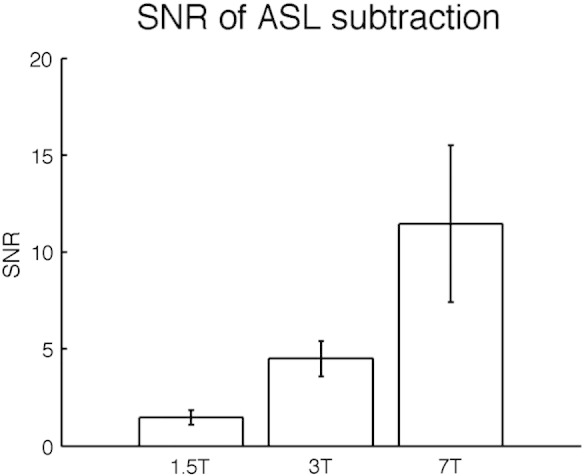
Signal to noise ratios of subtracted ASL images (tag-control) at different field strengths during the baseline condition. Signal was measured in motor regions, defined by BOLD/GM ROIs (prior to the removal of noisy voxels). Error bars show ± one standard deviation.

Finally, Fig. 7 shows how the SNR of resting ASL data increases with field strength. This is generally considered to be the limiting factor on the accuracy of calibrated BOLD methods, as the ASL signal – the difference between tag and control images – is always substantially smaller than the directly measured BOLD signal.

## Discussion

Measurements of stimulus evoked changes in CMRO_2_ using gas calibrated fMRI provide a more direct physiological measurement of underlying neural activity than standard BOLD fMRI. The technique was developed at 1.5 T and has increased in popularity with the more widespread availability of 3 T systems. However, very little research has been published to date regarding the translation of the technique to 7 T. Simulations predict that the Davis model parameters must be adjusted for this increase in field strength by using a *β* value of 1, compared with 1.5 and 1.3 at field strengths of 1.5 T and 3 T (Blockley et al., 2013; Griffeth et al., 2013). The small number of published experimental 7 T studies have universally used *β* = 1, consistent with these simulations (Driver et al., 2012; Hall et al., 2012; Krieger et al., 2014). The potential advantages of performing calibrated fMRI at 7 T come from improved image SNR for both BOLD and ASL (Triantafyllou et al., 2005) and an increase in longitudinal relaxation times for ASL resulting in higher SNR CBF estimates (Franke et al., 2000). This increased SNR has so far been used to produce maps of M (Hall et al., 2012) and to incorporate per-subject measurements of changes in CBV, rather than assume a fixed CBF–CBV coupling (Driver et al., 2012). However, the accuracy of CMRO_2_ measurements made at 7 T has until now been untested. In this study the validity of the calibrated fMRI technique across field strengths was investigated by examining the consistency of measurements acquired at 1.5 T, 3 T and 7 T.

### Main findings

The relative changes in CBF and CMRO_2_ in response to a motor task observed in this study are broadly in line with those reported in the literature. For example, Donahue et al. (2009) reported a CBF increase of 46 ± 11% and CMRO_2_ increase of 12 ± 13% at 3 T; Kastrup et al. (2002) reported a CBF increase of 71 ± 9% and a CMRO_2_ increase of 16 ± 9% at 1.5 T; Petr et al. (2014) reported a group average CBF increase of 47.2% at 3 T; Stefanovic et al. (2006) reported a CBF change of 45.6 ± 0.57% at 1.5 T; and Vilela et al. (2011) reported a CBF change of 62 ± 7% at 3 T. All of these studies also used pulsed ASL techniques.

Estimates of M were observed to increase roughly linearly with field strength (Fig. 4), consistent with the results of the detailed BOLD signal model simulations (Fig. 2). M values at 7 T were found to have the largest standard deviation, despite the predicted improvements in SNR (Table 2). Simulations indicate that variations in the underlying cerebral physiology (such as CBV and OEF) will result in a broader range of M values as magnetic field increases. This suggests that the increase in the standard deviation of M at higher fields is due to differences in physiology across the subject group, rather than an increase in random noise. This ability of M to control for physiological variability is an important characteristic of the Davis model, meaning that this variability is not propagated through to estimates of CMRO_2_ change.

Intra-subject CMRO_2_ was consistent between 3 and 7 T, which is encouraging for research centres investing in ultra-high field scanners. Future studies at these high fields could make use of the improved SNR to image at a higher resolution than has been possible until now. Changes in CMRO_2_ measured at 1.5 T were consistently greater than those at 3 and 7 T (Figs. 4 and 5). We hypothesize that this is an artefact of the lower SNR of 1.5 T scanners (see Fig. 7) combined with our voxel inclusion criteria for ROI analysis, which together artificially increase the average CBF response of the remaining voxels for analysis at 1.5 T as compared to 3 and 7 T. As Fig. 6 shows, the distribution of motor responses is much broader at 1.5 T than at higher field strengths, elevating the mean of remaining voxels.

The exclusion of approximately 50% of voxels from further analysis within the BOLD/GM ROI at 1.5 T is a cause for concern. In experiments carried out at either 3 or 7 T, ROIs are typically defined from voxels that exhibit positive BOLD and ASL responses (either in absolute terms or those with the highest z-stats) to tasks, and there is no need to define an additional “GM” criterion. Unfortunately due to the poor SNR of pulsed ASL data, especially at 1.5 T, this was not viable in the current study and it was necessary to rely only on BOLD and resting ASL data for ROI determination. Fig. 6 highlights the difficulties when using pulsed ASL at 1.5 T, demonstrating that the high level of noise can introduce significant biases during analysis, and that these results should be interpreted with caution.

### ROI selection

The large physiological and inherent scanner noise present in functional data, especially in ASL, presents a challenge. Increasing voxel size helps to increase SNR, but at the cost of lower specificity and greater partial voluming within voxels. This can make it difficult to create accurate GM masks, as many voxels will contain a mix of tissue types. In addition, the small number of slices and large voxels acquired in the current study made accurate co-registration of functional to structural data impossible. As a surrogate, good tag-control contrast in resting ASL data (i.e. high baseline perfusion) was used to define GM voxels (Gauthier and Hoge, 2013).

The choice of ROI – in this case, BOLD only versus BOLD/GM – had a significant impact on the ensuing estimates of M. Values calculated within the BOLD only motor ROI were comparable with results from previous studies at 3 T, at which the majority of calibrated fMRI studies have been carried out (see Table 3). M values in the BOLD/GM ROI were larger, but were in closer agreement with the predictions of the simulations. This is likely a reflection on the assumptions of the detailed BOLD signal model used for simulations. In contrast the method used to create the BOLD only ROI intentionally followed the analysis procedure from past studies rather than matching modelling assumptions. Recent studies at 7 T have taken greater care to extract only GM voxels for analysis, with methods and resulting M values more similar to our BOLD/GM ROI. Studies at 3 T have also recommended defining ROIs based on mapping stimulus evoked changes in CBF, which have been shown to result in greater reproducibility of CMRO_2_ estimates across sessions (Leontiev and Buxton, 2007). Defining the ROI in this manner is also likely to more closely align with the assumptions of the detailed BOLD signal model. However, our ability to apply this technique in this study was limited by the SNR of the ASL measurements, particularly at 1.5 T.

Because of the wide distribution of CBF changes within both ROIs, it was necessary to exclude some further voxels from later analysis. Thresholds were applied to include only those voxels with relative changes in CBF between 1 and 3 (in response to motor or hypercapnia stimuli) in order to remove voxels that were deemed unacceptably noisy or contained an insufficient fraction of grey matter. However, as the remaining voxels do not even approximate a normal distribution (see Fig. 6), neither the mean nor median values truly capture the overall flow response in the ROI. Nonetheless we followed convention and used mean values for further analysis, but this caused a noticeably higher apparent blood flow responses at 1.5 T as compared to 3 and 7 T (see Table 2).

The results of this study suggest that the Davis model is reassuringly insensitive to field strength, provided that the value for *β* is adjusted appropriately (Bulte et al., 2009; Davis et al., 1998; Driver et al., 2012). The observation that choice of ROI has a large effect on M but only a minimal impact on CMRO_2_ is a reflection of the power of the model in regressing out potentially confounding parameters. By combining all auxiliary parameters into one calibration constant, M, the model becomes very wide-ranging and remains relatively insensitive to residual factors, at least within healthy tissue.

### Limitations

The primary question that this study sought to answer was whether the translation of calibrated fMRI from 3 to 7 T would alter the estimated changes in CMRO_2_ during a functional task. In an attempt to remove confounding factors from this comparison, scan parameters were kept constant wherever possible. This included implementing the same pulse sequence (FAIR) with the same readout (single echo EPI at 17 ms). Unfortunately the use of a single echo was not feasible at 1.5 T, where the optimal BOLD echo time is much longer (50 ms or more), so it was necessary to modify the sequence and add a second echo at this lower field strength. As a result of these choices, readouts were acquired at close to optimal BOLD echo times at 1.5 T (50 ms, optimal is 50 ms) and 7 T (17 ms, optimal is 25 ms), whereas the 3 T BOLD signal was extracted from the 17 ms echo despite a longer optimal TE of 35 ms. This discrepancy led to a lower experimental BOLD response at 3 T (see Table 2), and may have negatively impacted the BOLD SNR at this field strength. The lower BOLD value was accounted for by the linear scaling of M value to the optimal echo time (see Methods section), although of course this cannot recover the lost SNR.

SNR is inherently lower at 1.5 T compared with higher field strengths. This problem has been amplified in this study by the use of a pulsed ASL sequence and a 12-channel head coil. In comparison to (pseudo-)continuous ASL, pulsed ASL is more sensitive to changes in flow velocity during motor tasks and gas challenges, as shortened arterial arrival times during stimulation may lead to differing volumes of tagged blood arriving at the imaging planes. Furthermore, the increased intravascular contribution to the BOLD signal at lower field strengths may further contribute to the CMRO_2_ discrepancy; in the absence of large vessels, ~ 57% of all signal at 1.5 T is of intravascular origin, as compared to ~ 36% at 3 T and a negligible contribution at 7 T (Uludag et al., 2009). However, it has been shown that the Davis model can account for this signal contribution via the parameters *α* and *β* (Griffeth and Buxton, 2011).

Another consequence of the use of a pulsed ASL sequence is the potential for underestimating flow responses during hypercapnia. Tancredi et al. (2012) have reported that although pulsed and pseudo-continuous methods show good agreement in baseline perfusion and focal activation responses, the global nature of the CBF increase during hypercapnia appears to lead to an underestimation in flow response when pulsed ASL is used. This may have led to an overestimation in M values and thus in relative CMRO_2_ estimates during motor tasks. However, this issue would have impacted all fields equally, and thus is not expected to affect the conclusions of this study.

In order to implement the same protocol on all three scanners, it was necessary to make several compromises in terms of sequence design and choice of parameters. It is important to bear in mind that SNR at 1.5 and 3 T could be improved by implementing a pseudo-continuous ASL sequence; similarly the intrinsically greater SNR at 7 T could be utilized by increasing the resolution, which would also reduce physiological noise (Triantafyllou et al., 2005). However, the primary aim of this work was to fairly compare the outcomes of the Davis model as a function of field strength only, by keeping as many other variables constant as possible.

## Conclusion

Changes in CMRO_2_ during a motor task, as calculated by the Davis model, were consistent between 3 and 7 T and were also in close agreement with the results of theoretical simulations. This is encouraging for future studies of calibrated fMRI at ultra-high fields, and supports the continued use of this simple signal model. The lower SNR at 1.5 T may present problems for the calibrated fMRI method, which relies heavily on ASL data. In this study the CMRO_2_ results were consistently over-estimated at 1.5 T, although this may be a result of the sub-optimal pulsed ASL sequence used. Voxel exclusion criteria and methods for ROI creation for post-processing are important and should be carefully considered and clearly stated in future studies, as they can have a significant impact on results.

## Figures and Tables

**Table 1:** Simulation parameters that change with field strength.

Variable	1.5 T	3 T	7 T	Description
*C*_1_	7.2 s^− 1^	13.8 s^− 1^	75.2 s^− 1^	Constant term in quadratic dependence of intravascular *R*_2_* on oxygenation (Blockley et al., 2008)
*C*_2_	95.1 s^− 1^	276.0 s^− 1^	831.9 s^− 1^	Quadratic term in quadratic dependence of intravascular *R*_2_* on oxygenation (Blockley et al., 2008)
*R*_2E_*(0)	11.6 s^− 1^	18.1 s^− 1^	30.8 s^− 1^	Resting extravascular rate of signal decay (van der Zwaag et al., 2009)
TE	50 ms	17 ms	17 ms	BOLD echo time

**Table 2:** Summary of results.

		1.5 T	3 T	7 T
BOLD only ROI	ΔBOLD to CO_2_	0.017 ± 0.005	0.010 ± 0.002	0.017 ± 0.006
	ΔBOLD to motor	0.012 ± 0.003	0.007 ± 0.002	0.014 ± 0.004
	CBF to CO_2_	1.34 ± 0.05	1.24 ± 0.05	1.24 ± 0.07
	CBF to motor	1.58 ± 0.08	1.41 ± 0.05	1.42 ± 0.07
	M	0.055 ± 0.015	0.051 ± 0.011	0.115 ± 0.044
	M at optimal TE	0.055 ± 0.015	0.105 ± 0.023	0.169 ± 0.064
	CMRO_2_ to motor	1.26 ± 0.08	1.19 ± 0.06	1.13 ± 0.07
	Voxels analyzed	118 ± 41	197 ± 61	204 ± 88
BOLD/GM ROI	ΔBOLD to CO_2_	0.018 ± 0.005	0.009 ± 0.002	0.016 ± 0.006
	ΔBOLD to motor	0.013 ± 0.003	0.006 ± 0.001	0.014 ± 0.004
	CBF to CO_2_	1.20 ± 0.05	1.13 ± 0.03	1.12 ± 0.03
	CBF to motor	1.47 ± 0.08	1.31 ± 0.05	1.33 ± 0.05
	M	0.088 ± 0.024	0.072 ± 0.012	0.186 ± 0.079
	M at optimal TE	0.088 ± 0.024	0.149 ± 0.026	0.274 ± 0.116
	CMRO_2_ to motor	1.25 ± 0.07	1.17 ± 0.05	1.15 ± 0.04
	Voxels analyzed	61 ± 23	95 ± 35	94 ± 44

Summary of experimental results, given as mean ± standard deviation. CBF and CMRO_2_ have been normalized to baseline values (no change = 1), and ΔBOLD and M are given as fractional changes with respect to baseline (no change = 0). Optimal echo time (TE) is 50/35/25 ms at 1.5/3/7 T respectively.

**Table 3:** Comparison of M values in the current study with those in the literature.

Field	Study	ROI	M (at optimal TE)
1.5 T	**Current study (BOLD only ROI)**	**Motor**	**0.055 ± 0.015**
	Davis et al. (1998)	Visual	0.056 ± 0.017
	Stefanovic et al. (2006)	Motor	0.061 ± 0.011
	Stefanovic et al. (2004)	Motor	0.072 ± 0.010
	**Current study (BOLD/GM ROI)**	**Motor**	**0.088 ± 0.024**
	Kastrup et al. (2002)	Motor	0.113 ± 0.038
	Hoge et al. (1999)	Visual (single)Visual (graded)	0.15 ± 0.06 0.22 ± 0.03
3 T	Chiarelli et al. (2007a)	Motor	0.047 ± 0.038
	Chen and Parrish (2009)	Motor	0.056 ± 0.015
	Ances et al. (2008)	Visual	0.067 ± 0.002
	Chiarelli et al. (2007b)	Motor	0.069 ± 0.006 (oxygen)
	Mark et al. (2011)	Motor	0.081 ± 0.011
	Ances et al. (2009)	Visual	0.085 ± 0.006
	**Current study (BOLD only ROI)**	**Motor**	**0.105 ± 0.023**
	Gauthier et al. (2011)	Visual	0.111 (carbogen)
	Lin et al. (2008)	Visual	0.123 ± 0.003
	Leontiev and Buxton (2007)	Visual	0.130 ± 0.069
	Perthen et al. (2008)	Visual	0.141 ± 0.013
	**Current study (BOLD/GM ROI)**	**Motor**	**0.149 ± 0.026**
7 T	**Current study (BOLD only ROI)**	**Motor**	**0.169 ± 0.064**
	**Current study (BOLD/GM ROI)**	**Motor**	**0.274 ± 0.116**
	Driver et al. (2012)	Motor	0.28 ± 0.02 (oxygen)
	Hall et al. (2012)	Motor	0.29 ± 0.04
	Krieger et al. (2014)	Motor	0.309 ± 0.031 (carbogen)

All studies used hypercapnia unless indicated otherwise. Note that all M values and errors have been linearly scaled to the optimal echo times of 50/35/25 ms for 1.5/3/7 T respectively.

## References

[bb0005] Alsop D.C., Detre J.A., Golay X., Gunther M., Hendrikse J., Hernandez-Garcia L., Lu H., Macintosh B.J., Parkes L.M., Smits M., van Osch M.J., Wang D.J., Wong E.C., Zaharchuk G. (2015). Recommended implementation of arterial spin-labeled perfusion MRI for clinical applications: a consensus of the ISMRM perfusion study group and the European consortium for ASL in dementia. Magn. Reson. Med..

[bb0010] Ances B.M., Leontiev O., Perthen J.E., Liang C., Lansing A.E., Buxton R.B. (2008). Regional differences in the coupling of cerebral blood flow and oxygen metabolism changes in response to activation: implications for BOLD-fMRI. NeuroImage.

[bb0015] Ances B.M., Liang C.L., Leontiev O., Perthen J.E., Fleisher A.S., Lansing A.E., Buxton R.B. (2009). Effects of aging on cerebral blood flow, oxygen metabolism, and blood oxygenation level dependent responses to visual stimulation. Hum. Brain Mapp..

[bb0025] Blockley N.P., Jiang L., Gardener A.G., Ludman C.N., Francis S.T., Gowland P.A. (2008). Field strength dependence of R1 and R2* relaxivities of human whole blood to ProHance, Vasovist, and deoxyhemoglobin. Magn. Reson. Med..

[bb0020] Blockley N.P., Griffeth V.E.M., Jezzard P., Bulte D.P. (2013). Cross-field Analysis of Hypercapnia Calibrated BOLD.

[bb0030] Bulte D.P., Drescher K., Jezzard P. (2009). Comparison of hypercapnia-based calibration techniques for measurement of cerebral oxygen metabolism with MRI. Magn. Reson. Med..

[bb0045] Chen Y., Parrish T.B. (2009). Caffeine's effects on cerebrovascular reactivity and coupling between cerebral blood flow and oxygen metabolism. NeuroImage.

[bb0035] Chen J.J., Pike G.B. (2009). BOLD-specific cerebral blood volume and blood flow changes during neuronal activation in humans. NMR Biomed..

[bb0040] Chen J.J., Pike G.B. (2010). Global cerebral oxidative metabolism during hypercapnia and hypocapnia in humans: implications for BOLD fMRI. J. Cereb. Blood Flow Metab..

[bb0050] Chiarelli P.A., Bulte D.P., Gallichan D., Piechnik S.K., Wise R., Jezzard P. (2007). Flow-metabolism coupling in human visual, motor, and supplementary motor areas assessed by magnetic resonance imaging. Magn. Reson. Med..

[bb0055] Chiarelli P.A., Bulte D.P., Wise R., Gallichan D., Jezzard P. (2007). A calibration method for quantitative BOLD fMRI based on hyperoxia. NeuroImage.

[bb0060] Davis T.L., Kwong K.K., Weisskoff R.M., Rosen B.R. (1998). Calibrated functional MRI: mapping the dynamics of oxidative metabolism. Proc. Natl. Acad. Sci. U. S. A..

[bb0065] Donahue M.J., Blicher J.U., Ostergaard L., Feinberg D.A., MacIntosh B.J., Miller K.L., Gunther M., Jezzard P. (2009). Cerebral blood flow, blood volume, and oxygen metabolism dynamics in human visual and motor cortex as measured by whole-brain multi-modal magnetic resonance imaging. J. Cereb. Blood Flow Metab..

[bb0070] Driver I.D., Hall E.L., Wharton S.J., Pritchard S.E., Francis S.T., Gowland P.A. (2012). Calibrated BOLD using direct measurement of changes in venous oxygenation. NeuroImage.

[bb0075] Franke C., van Dorsten F.A., Olah L., Schwindt W., Hoehn M. (2000). Arterial spin tagging perfusion imaging of rat brain: dependency on magnetic field strength. Magn. Reson. Imaging.

[bb0080] Gardener A.G., Gowland P.A., Francis S.T. (2009). Implementation of quantitative perfusion imaging using pulsed arterial spin labeling at ultra-high field. Magn. Reson. Med..

[bb0085] Gauthier C.J., Hoge R.D. (2013). A generalized procedure for calibrated MRI incorporating hyperoxia and hypercapnia. Hum. Brain Mapp..

[bb0090] Gauthier C.J., Madjar C., Tancredi F.B., Stefanovic B., Hoge R.D. (2011). Elimination of visually evoked BOLD responses during carbogen inhalation: implications for calibrated MRI. NeuroImage.

[bb0100] Griffeth V.E., Buxton R.B. (2011). A theoretical framework for estimating cerebral oxygen metabolism changes using the calibrated-BOLD method: modeling the effects of blood volume distribution, hematocrit, oxygen extraction fraction, and tissue signal properties on the BOLD signal. NeuroImage.

[bb0095] Griffeth V.E., Blockley N.P., Simon A.B., Buxton R.B. (2013). A new functional MRI approach for investigating modulations of brain oxygen metabolism. PLoS One.

[bb0105] Grubb R.L., Raichle M.E., Eichling J.O., Ter-Pogossian M.M. (1974). The effects of changes in PaCO2 on cerebral blood volume, blood flow, and vascular mean transit time. Stroke.

[bb0110] Hall E.L., Driver I.D., Pritchard S.E., Gowland P.A., Francis S.T. (2012). Voxel-wise Estimation of M and CMRO2 at 7 T.

[bb0115] Hatazawa J., Fujita H., Kanno I., Satoh T., Iida H., Miura S., Murakami M., Okudera T., Inugami A., Ogawa T. (1995). Regional cerebral blood flow, blood volume, oxygen extraction fraction, and oxygen utilization rate in normal volunteers measured by the autoradiographic technique and the single breath inhalation method. Ann. Nucl. Med..

[bb0120] Hoge R.D. (2012). Calibrated FMRI. NeuroImage.

[bb0125] Hoge R.D., Atkinson J., Gill B., Crelier G.R., Marrett S., Pike G.B. (1999). Investigation of BOLD signal dependence on cerebral blood flow and oxygen consumption: the deoxyhemoglobin dilution model. Magn. Reson. Med..

[bb0130] Jenkinson M., Bannister P., Brady M., Smith S. (2002). Improved optimization for the robust and accurate linear registration and motion correction of brain images. NeuroImage.

[bb0135] Jenkinson M., Beckmann C.F., Behrens T.E., Woolrich M.W., Smith S.M. (2012). Fsl. NeuroImage.

[bb0140] Kastrup A., Kruger G., Neumann-Haefelin T., Glover G.H., Moseley M.E. (2002). Changes of cerebral blood flow, oxygenation, and oxidative metabolism during graded motor activation. NeuroImage.

[bb0145] Kim S.G. (1995). Quantification of relative cerebral blood flow change by flow-sensitive alternating inversion recovery (FAIR) technique: application to functional mapping. Magn. Reson. Med..

[bb0150] Krieger S.N., Gauthier C.J., Ivanov D., Huber L., Roggenhofer E., Sehm B., Turner R., Egan G.F. (2014). Regional reproducibility of calibrated BOLD functional MRI: implications for the study of cognition and plasticity. NeuroImage.

[bb0155] Kriegeskorte N., Simmons W.K., Bellgowan P.S., Baker C.I. (2009). Circular analysis in systems neuroscience: the dangers of double dipping. Nat. Neurosci..

[bb0160] Leontiev O., Buxton R.B. (2007). Reproducibility of BOLD, perfusion, and CMRO2 measurements with calibrated-BOLD fMRI. NeuroImage.

[bb0165] Lin A.L., Fox P.T., Yang Y., Lu H., Tan L.H., Gao J.H. (2008). Evaluation of MRI models in the measurement of CMRO2 and its relationship with CBF. Magn. Reson. Med..

[bb0170] Mark C.I., Fisher J.A., Pike G.B. (2011). Improved fMRI calibration: precisely controlled hyperoxic versus hypercapnic stimuli. NeuroImage.

[bb0175] McPhee S.J., Hammer G.D. (2009). Pathophysiology of Disease an Introduction to Clinical Medicine.

[bb0180] Ogawa S., Menon R.S., Tank D.W., Kim S.G., Merkle H., Ellermann J.M., Ugurbil K. (1993). Functional brain mapping by blood oxygenation level-dependent contrast magnetic resonance imaging. A comparison of signal characteristics with a biophysical model. Biophys. J..

[bb0185] Perlmutter J.S., Powers W.J., Herscovitch P., Fox P.T., Raichle M.E. (1987). Regional asymmetries of cerebral blood flow, blood volume, and oxygen utilization and extraction in normal subjects. J. Cereb. Blood Flow Metab..

[bb0190] Perthen J.E., Lansing A.E., Liau J., Liu T.T., Buxton R.B. (2008). Caffeine-induced uncoupling of cerebral blood flow and oxygen metabolism: a calibrated BOLD fMRI study. NeuroImage.

[bb0195] Petr J., Ferre J.C., Raoult H., Bannier E., Gauvrit J.Y., Barillot C. (2014). Template-based approach for detecting motor task activation-related hyperperfusion in pulsed ASL data. Hum. Brain Mapp..

[bb0200] Smith S.M. (2002). Fast robust automated brain extraction. Hum. Brain Mapp..

[bb0205] Stefanovic B., Warnking J.M., Pike G.B. (2004). Hemodynamic and metabolic responses to neuronal inhibition. NeuroImage.

[bb0210] Stefanovic B., Warnking J.M., Rylander K.M., Pike G.B. (2006). The effect of global cerebral vasodilation on focal activation hemodynamics. NeuroImage.

[bb0215] Tancredi F.B., Gauthier C.J., Madjar C., Bolar D.S., Fisher J.A., Wang D.J., Hoge R.D. (2012). Comparison of pulsed and pseudocontinuous arterial spin-labeling for measuring CO_2_-induced cerebrovascular reactivity. J. Magn. Reson. Imaging.

[bb0220] Triantafyllou C., Hoge R.D., Krueger G., Wiggins C.J., Potthast A., Wiggins G.C., Wald L.L. (2005). Comparison of physiological noise at 1.5 T, 3 T and 7 T and optimization of fMRI acquisition parameters. NeuroImage.

[bb0225] Uludag K., Muller-Bierl B., Ugurbil K. (2009). An integrative model for neuronal activity-induced signal changes for gradient and spin echo functional imaging. NeuroImage.

[bb0230] van der Zwaag W., Francis S., Head K., Peters A., Gowland P., Morris P., Bowtell R. (2009). fMRI at 1.5, 3 and 7 T: characterising BOLD signal changes. NeuroImage.

[bb0235] Vilela P., Pimentel M., Sousa I., Figueiredo P. (2011). Quantification of perfusion changes during a motor task using arterial spin labeling. Neuroradiol. J..

[bb0240] Wong E.C., Buxton R.B., Frank L.R. (1998). Quantitative imaging of perfusion using a single subtraction (QUIPSS and QUIPSS II). Magn. Reson. Med..

[bb0245] Woolrich M.W., Jbabdi S., Patenaude B., Chappell M., Makni S., Behrens T., Beckmann C., Jenkinson M., Smith S.M. (2009). Bayesian analysis of neuroimaging data in FSL. NeuroImage.

[bb0250] Zhao J.M., Clingman C.S., Narvainen M.J., Kauppinen R.A., van Zijl P.C. (2007). Oxygenation and hematocrit dependence of transverse relaxation rates of blood at 3 T. Magn. Reson. Med..

